# Outcomes of stereotactic body radiotherapy 60 Gy in 8 fractions when prioritizing organs at risk for central and ultracentral lung tumors

**DOI:** 10.1186/s13014-020-01491-w

**Published:** 2020-02-27

**Authors:** Yizhou Zhao, Eman Khawandanh, Steven Thomas, Susan Zhang, Emma M. Dunne, Mitchell Liu, Devin Schellenberg

**Affiliations:** 1 Department of Radiation Oncology, BC Cancer Surrey, 13750 96 Ave, Surrey, BC V3V 1Z2 Canada; 2 Present Address: Department of Radiation Oncology, CHU de Québec, 11 Côte du Palais, Quebec, QC G1R 2J6 Canada; 3 Department of Medical Physics, BC Cancer Vancouver, 600 W 10th Ave, Vancouver, BC V5Z 4E6 Canada; 4 Department of Radiation Oncology, BC Cancer Vancouver, 600 W 10th Ave, Vancouver, BC V5Z 4E6 Canada

**Keywords:** Stereotactic body radiotherapy, Central, Ultracentral, Lung tumors, 60 Gy in 8 fractions

## Abstract

**Background:**

For stereotactic body radiotherapy (SBRT) to central (C) and ultracentral (UC) lung tumors, our provincial practice has been to prioritize organs at risk (OARs) constraints by compromising target volume coverage if needed. The objectives are to report the treatment’s efficacy and safety.

**Methods:**

We conducted a retrospective analysis of all provincial patients who underwent SBRT at 60Gy in 8 fractions to C and UC lung tumors, from 2013 to 2017.

**Results:**

Ninety-eight lesions were treated, 57 (58.2%) C and 41 (41.8%) UC. The median follow-up was 22.9 months (range 2.5–64.8 months). The 1- and 3-year local control (LC) was 97.8 and 84.5% respectively, with no differences between C and UC groups (*p* = 0.662). Fifty-three (54.1%) cases had optimal dose coverage (V60Gy ITV&PTV > 95%), 29 (29.6%) had compromised PTV coverage (V60Gy ITV > 95%/PTV < 95%), and 16 (16.3%) had both compromised ITV and PTV coverage (V60Gy ITV&PTV < 95%). No significant difference in LC was detected at 2 years between the 3 groups (95.6, 91.8 and 90.9%, *p* = 0.717). There were 3 episodes of grade 3 toxicity in the C group (2 dyspnea, 1 pneumonitis) and 2 in the UC group (1 dyspnea, 1 hemoptysis). There were no gr4/5 toxicities. On multivariable Cox regression analysis, ITV size was found to be a predictor for LC (*p* = 0.001).

**Conclusions:**

SBRT at 60Gy in 8 fractions achieves high rates of LC with low risks of significant toxicities, even if target volume coverage is reduced to meet OARs constraints.

## Background

Stereotactic body radiotherapy (SBRT) is widely accepted as the standard treatment for early stage non-small cell lung cancers (NSCLC) in patients who are not operative candidates [1–3]. The applications of thoracic SBRT has expanded over time and it is now increasingly used for the ablative treatment of metastatic lesions, particularly in the settings of oligometastatic and oligoprogressive disease [4–7].

Excellent local control rates can be achieved for early stage NSCLC treated with SBRT, with a landmark study by Timmerman et al. demonstrating a 3-year local control rate of 97.6% [2]. However, an early phase II trial reported risks of severe treatment-related toxicities for a subset of lesions located centrally in the chest [8]. The 2-year freedom from severe toxicity rate was only 54% for central tumors, in contrast to 83% for those that are peripherally situated. Thus, a central at risk region consisting of 2 cm expansion around the proximal bronchial tree has traditionally been labelled as the ‘no-fly zone.’ [8] More recently, the concept of ultracentral lung tumors has emerged, referring to a higher risk subgroup of central lung tumors. Although there is no universally accepted definition for an ultracentral lesion, it is generally considered as a lesion with gross disease or planning target volume (PTV) overlapping the proximal bronchial tree and/or critical mediastinal structures [9, 10].

At present, there is no consensus regarding the optimal dose fractionation for SBRT to centrally and ultracentrally located lung tumors. Nonetheless, it is recommended to use a protracted fractionation regimen to minimize the risks of treatment-related complications [11]. It is also not known whether one should prioritize target volume coverage or organs at risk (OARs) safety, when a compromise is needed. Thus, the SBRT treatment of central and ultracentral lung lesions remains controversial and the pattern of practice is variable among different institutions [12].

The protocol across the 6 cancer centres of our province has been to treat all centrally and ultracentrally located lung lesions with 60 Gy in 8 fractions. When required, our general practice has been to compromise target volumes coverage to minimize excessive dose to critical OARs. This study reports our experience and the outcomes of our patients who underwent SBRT for central and ultracentral lung tumors.

## Methods

### Patient inclusion criteria

This study received the full approval from the institutional research ethics board. Central lung tumors are defined as lesions located within 2 cm of the proximal bronchial tree or with planning target volume (PTV) overlapping the mediastinal and/or pericardial pleura, as per the criteria of the Radiation Therapy Oncology Group (RTOG) 0813 study [13]. A tumor is considered ultracentral if its PTV overlaps the proximal bronchial tree, esophagus, pulmonary vein or pulmonary artery, consistent with the definition used in the ongoing SUNSET study [14].

In our province, all SBRT treatments for central and ultracentral lung tumors were delivered at a dose of 60 Gy in 8 fractions. All patients who underwent treatment to a central or an ultracentral lung tumor from the start of our central lung SBRT program in January 2013 to December 2017 were identified and included in this study. Both biopsy-confirmed and presumed primary lung malignancies and metastatic lesions of any histology were included. The cases with a single PTV that incorporated more than 1 lesion were excluded.

### Stereotactic body radiotherapy treatment

For purposes of staging, computed tomography (CT) and positron emission tomography (PET)-CT imagings were performed. All patients underwent a 4-dimensional computed tomography (4DCT) simulation and were treated with motion encompassing technique. Respiratory gating, breath-hold and dynamic tumor tracking methods were not used. Target volumes, OARs delineation, and treatment planning were performed on the average phase of the 4DCT. The gross tumor volume (GTV) was defined as the pulmonary lesion seen on the average phase of the 4DCT, delineated with aid of all the available diagnostic imaging. An internal target volume (ITV) was generated non-isotropically, taking into account the GTV motion on all respiratory phases of the 4DCT. PTV was ITV with a 5 mm isotropic expansion. OARs were contoured in full and included the proximal bronchial tree, proximal trachea, esophagus, heart, great vessels, bilateral lungs, chest wall, spinal cord and ipsilateral brachial plexus. For the purpose of OAR delineation, the proximal bronchial tree included the carina, right and left main bronchi, right and left upper lobe bronchi, intermedius bronchus, lingular bronchus, right and left lower lobe bronchi.

A dose of 60 Gy in 8 fractions was prescribed to the 90% isodose line. The planning aims were to cover at least 95% of the PTV volume by the prescription dose (V 100% > 95%) and to cover at least 99% of the PTV volume by 54 Gy (V 90% > 99%). Our provincial dose constraint guidelines were used (Appendix A). Full target volumes coverage for SBRT treatment of central and ultracentral lung lesions can lead to significant overdosage of critical OARs, particularly in situations of overlap with the target volumes. While exceeding the established provincial OARs dose constraints is allowed at the discretion of the treating physician, the pattern of practice across our institution has been to compromise target volumes coverage to minimize excessive dose to the critical OARs.

The SBRT plans were generated using the Eclipse treatment planning system (Varian Medical Systems, Palo Alto, California, USA), with either 3-dimensional conformal radiotherapy (3DCRT), multifield intensity modulated radiotherapy (IMRT) or volumetric modulated arc therapy (VMAT) techniques. The planning technique was determined by the centres’ local practice at the time of treatment. 3DCRT technique was mainly used in the beginning of our lung SBRT program, and 5 out of the 6 provincial centres subsequently changed to IMRT and VMAT planning. The Analytical Anisotropic Algorithm (AAA) was used for dose calculations, with a dose grid of 0.2 cm. Treatments were delivered daily, over a period of 2 weeks. Kilovoltage (KV) orthogonal imaging and cone beam computed tomography (CBCT) were done prior to each radiotherapy fraction to ensure that the gross disease is properly situated inside the PTV at the time of treatment. Shifts were applied for any discrepancies.

### Patient follow-up

After the completion of SBRT, patients had chest CT scans and follow-up clinic assessments at intervals varying between 3 to 6 months in the first 5 years. The response evaluation criteria in solid tumors (RECIST) was used to assess for treatment response. Local failure was defined as disease progression within the 50% radiotherapy treatment isodose line. A confirmation of local disease recurrence by biopsy or PET scan was optional. Each case documented as local failure was assessed and confirmed by at least 2 thoracic radiation oncologists. SBRT related toxicities were also assessed at the time of each follow-up visit and were graded as per the common terminology criteria for adverse events (CTCAE) version 5.0 criteria.

### Data collection and statistical analysis

Clinical data up to the end of December 2018 were collected through a retrospective review of the patients’ medical records. Patient demographics (age, gender, eastern cooperative oncology group [ECOG] performance status, smoking status, medical comorbidities), tumor characteristics (location, histology, stage as per the 8th edition of the American joint committee on cancer classification system), clinical outcomes (events of local failure and death, with date if applicable) and CTCAE grade 3–5 treatment-related toxicities were collected. ITV and PTV dosimetric parameters were extracted from the Eclipse treatment planning system (size in cc, minimum dose [Dmin], maximum dose [Dmax], mean dose [Dmean], volume receiving at least 60 Gy [V 60 Gy], maximum dose covering 1% of the volume [D 1%], maximum dose covering 99% of the volume [D 99%], volume receiving < 60 Gy).

The statistical significance of differences in demographic and dosimetric parameters between the central and ultracentral lung tumors cohorts were determined using Pearson Chi-square test or Wilcoxon rank-sum test for categorical or continuous variables. Local control and overall survival were both calculated from the date of SBRT completion, using the Kaplan-Meier method. Since not all patients suffered an event during the time of follow-up, the data analysis was censored. Outcomes between different groups was compared using the log-rank test. For the local control outcomes, Cox proportional hazards regression analyses were performed to determine potential associations between patient, tumor and dosimetric parameters. All variables with a *p*-value less than 0.1 on the univariable analysis were candidate for inclusion in the multivariable analysis. All of the conducted tests are 2-tailed. The above analyses were performed with the Statistical Package for the Social Sciences version 25 (IBM Analytics, Armonk, New York).

## Results

### Patient and treatment characteristics

A total of 98 patients were included in this study. Fifty-seven (58.2%) had central and 41 (41.8%) had ultracentral lung lesions. There were 76 (77.6%) primary lung tumors (31 [40.8%] adenocarcinoma, 17 [22.4%] squamous cell carcinoma, 6 [7.9%] non-small cell lung carcinoma not otherwise specified, 22 [28.9%] presumed lung primaries without tissue diagnosis) and 22 (22.4%) metastases (8 [36.4%] primary colorectal, 4 [18.2%] sarcoma, 3 [13.6%] lung, 2 [9.1%] kidney, 2 [9.1%] prostate, 1 [4.5%] salivary gland, 1 [4.5%] melanoma, 1 [4.5%] neuroendocrine). The median follow-up time after the completion of SBRT was 22.9 months (range 2.5–64.8 months). The baseline patient and tumor characteristics are depicted in Table 1, with no significant differences between the central and ultracentral cohorts.

**Table 1:** Baseline Patient and Tumor Characteristics

Characteristics	All Patients *n* = 98	Central Tumors *n* = 57	Ultracentral tumors *n* = 41	*p*-value
Age at SBRT completion (years)				0.242
Median	74.0	75.2	71.7	
Range	43.6–89.6	55.4–89.6	43.6–85.3	
Gender				0.701
Male	42 (42.9%)	23 (40.4%)	19 (46.3%)	
Female	56 (57.1%)	34 (59.6%)	22 (53.7%)	
ECOG performance status				0.282
0	16 (16.3%)	11 (19.3%)	5 (12.2%)	
1	55 (56.1%)	34 (59.6%)	21 (51.2%)	
2	24 (24.5%)	10 (17.5%)	14 (34.1%)	
3	3 (3.1%)	2 (3.5%)	1 (2.4%)	
Smoking status				0.336
Active smoker	27 (27.6%)	18 (31.6%)	9 (22.0)	
Past smoker	56 (57.1%)	29 (50.9%)	27 (65.9%)	
Never smoker	15 (15.3%)	10 (17.5%)	5 (12.2%)	
COPD				0.249
GOLD stage 1	15 (15.3%)	12 (21.1%)	3 (7.3)	
GOLD stage 2	29 (29.6%)	13 (22.8%)	16 (39.0%)	
GOLD stage 3	13 (13.3%)	8 (14.0%)	5 (12.2%)	
GOLD stage 4	2 (2.0%)	1 (1.8%)	1 (2.4%)	
GOLD stage unknown	2 (2.0%)	2 (3.5%)	0 (0%)	
No COPD	37 (37.8%)	21 (36.8%)	16 (39.0%)	
Age-adjusted Charlson comorbidity index				0.942
Median	7	7	7	
Range	4–11	4–11	4–11	
Histology				0.378
Primary lung tumors	76 (77.6%)	46 (80.7%)	30 (73.2%)	
Metastases	22 (22.4%)	11 (19.7%)	11 (26.8%)	
T stage for primary lung tumors				0.304
T1a	2 (2.0%)	2 (3.5%)	0 (0%)	
T1b	25 (25.5%)	16 (28.1%)	9 (22.0%)	
T1c	25 (25.5%)	17 (29.8%)	8 (19.5%)	
T2a	17 (17.3%)	7 (12.3%)	10 (24.4%)	
T2b	4 (4.1%)	2 (3.5%)	2 (4.9%)	
T3	2 (2.0%)	2 (3.5%)	0 (0%)	
T4	1 (1.0%)	0 (0%)	1 (2.4%)	
Criteria to define central lung tumors			n/a	n/a
1 – GTV within 2 cm of proximal bronchial tree	24 (24.5%)	24 (42.1%)		
2 – PTV overlaps mediastinal / pericardial pleura	31 (31.6%)	31 (54.4%)		
1 and 2	2 (2.0%)	2 (3.5%)		
Criteria to define ultracentral lung tumors		n/a		n/a
1 – PTV overlaps proximal bronchial tree	26 (26.5%)		26 (63.4%)	
2 – PTV overlaps esophagus	1 (1.0%)		1 (2.4%)	
3 – PTV overlaps pulmonary artery	2 (2.0%)		2 (4.9%)	
4 – PTV overlaps pulmonary vein	2 (2.0%)		2 (4.9%)	
1 and 3	8 (8.2%)		8 (19.5%)	
1 and 4	1 (1.0%)		1 (2.4%)	
2 and 4	1 (1.0%)		1 (2.4%)	

*Abbreviations*: *SBRT* stereotactic body radiotherapy, *ECOG* eastern cooperative oncology group, *COPD* chronic obstructive pulmonary disease, *GOLD* global initiative for chronic obstructive lung disease, *GTV* gross tumor volume, *PTV* planning target volume

**Fig. 1:**
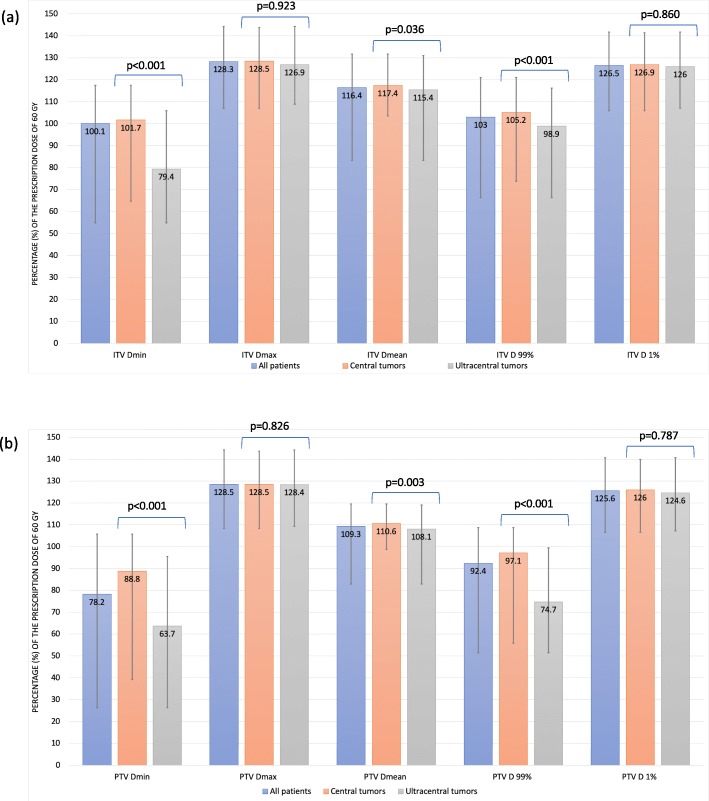
Median and range values of the dosimetric parameters for (**a**) internal target volume (ITV) and (**b**) planning target volume (PTV)

The median ITV size was 8.6 cc (range 0.6 cc – 71.8 cc) for central cases and 14.6 cc (range 1.0 cc – 106.1 cc) for ultracentral, with a statistically significant difference reflecting larger size for the ultracentral group (*p* = 0.005). The median PTV size was 25.7 cc (range 5.1 cc – 134.5 cc) for central cases and 42.1 cc (range 6.6 cc – 184.8 cc) for ultracentral, with a similar statistically significant difference (*p* = 0.002). The dosimetric parameters of the SBRT treatment plans are shown in Fig. 1. The median ITV V 60 Gy was 100.0% (range 85.3–100.0%) in the central group and 98.5% (range 22.1–100.0%) in the ultracentral group (*p* < 0.001). The median PTV V 60 Gy was 95.0% (range 70.8–100.0%) for the central group and 83.4% (range 20.6–98.3%) for the ultracentral group (*p* < 0.001). The median ITV volume receiving less than 60 Gy was 0 cc (range 0 cc – 9.9 cc) for the central group and 0.3 cc (range 0 cc – 12.9 cc) for the ultracentral group (*p* < 0.001). The median PTV volume receiving less than 60 Gy was 1.6 cc (range 0 cc – 38.0 cc) in the central group and 6.6 cc (range 0.5 cc – 67.8 cc) for the ultracentral group (*p* < 0.001). Overall better ITV and PTV dose coverage were achieved for the patients in the central tumor cohort, as reflected by significantly higher Dmin, Dmean, V 60 Gy and D 99%, as well as lower volumes receiving less than 60Gy.

### Tumor response

**Fig. 2:**
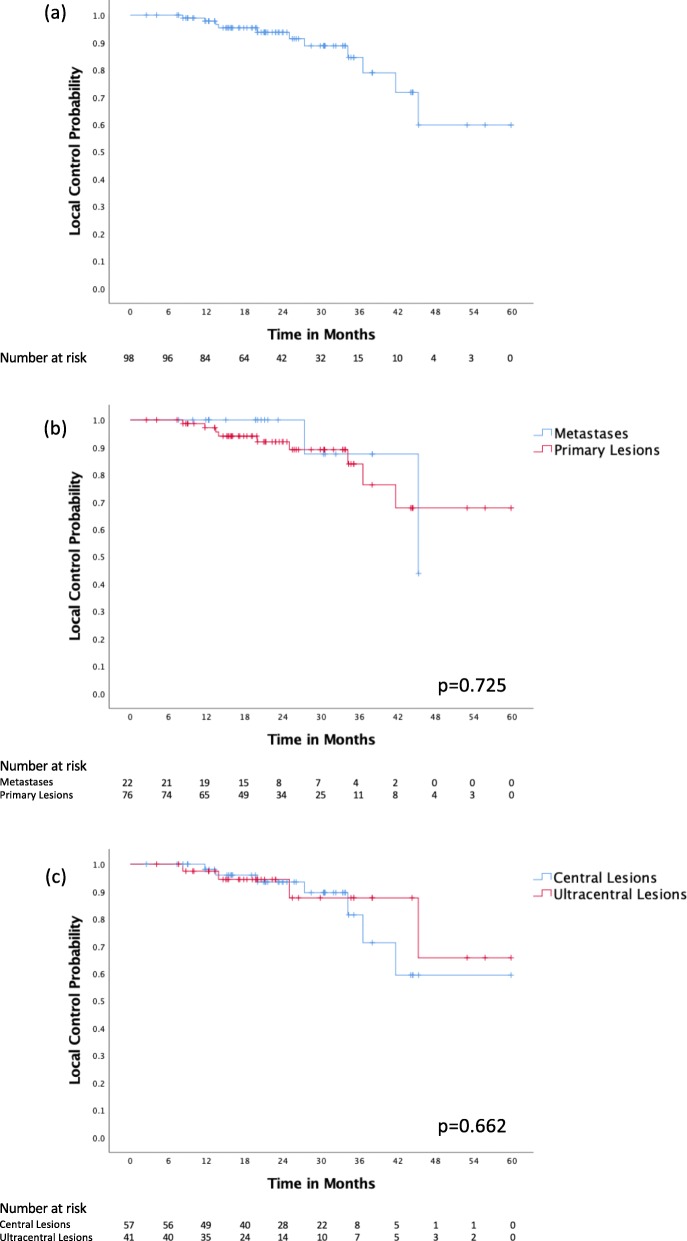
Local control for (**a**) all patients, (**b**) primary lesions vs metastases, (**c**) central vs ultracentral lesions

The local disease control outcomes are illustrated in Fig. 2. At the time of analysis, there were a total of 11 local failure events, 7 (63.6%) in the central group and 4 (36.4%) in the ultracentral group. Nine (81.8%) events occurred in patients treated for a primary lung lesion and 2 (18.2%) in those treated for a metastasis. For all patients in the study, the local control rates at 1 year, 2 years and 3 years were 97.8, 93.7 and 84.5% respectively. There was no significant difference in local control between primary lung lesions and metastases (*p* = 0.725), nor between central and ultracentral tumors (*p* = 0.662).

**Fig. 3:**
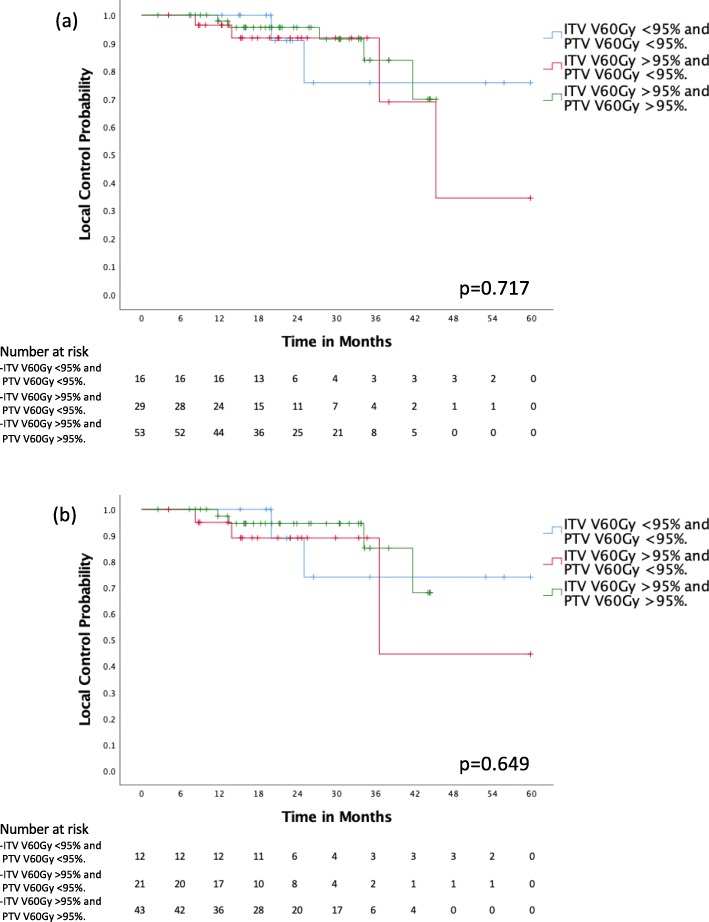
Local control per dose coverage for internal target volume (ITV) and planning target volume (PTV) for (**a**) all patients and (**b**) primary lung tumors

Fifty-three (54.1%) cases had optimal target volumes dose coverage, with ITV and PTV V 60 Gy > 95%. Twenty-nine (29.6%) patients had adequate ITV coverage but compromised PTV coverage, with ITV V 60 Gy ITV > 95% and PTV V 60 Gy < 95%. Sixteen (16.3%) patients had compromised ITV and PTV coverage, with ITV and PTV V 60 Gy < 95%. No statistically significant difference in local control was detected between the 3 groups, with rates at 2 years of 95.6, 91.8 and 90.9%. (*p* = 0.717) (Fig. 3). The same analysis was performed for the cohort of 76 (77.6%) patients with primary lung tumors. Forty-three (56.6%) patients had optimal ITV and PTV coverage, 21 (27.6%) with adequate ITV coverage but compromised PTV coverage and 12 (15.8%) with compromised ITV and PTV coverage. Similarly, there was no statistically significant difference in local control between the groups, with rates at 2 years of respectively 94.6%, 89.1 and 88.9% (*p* = 0.649).

**Fig. 4:**
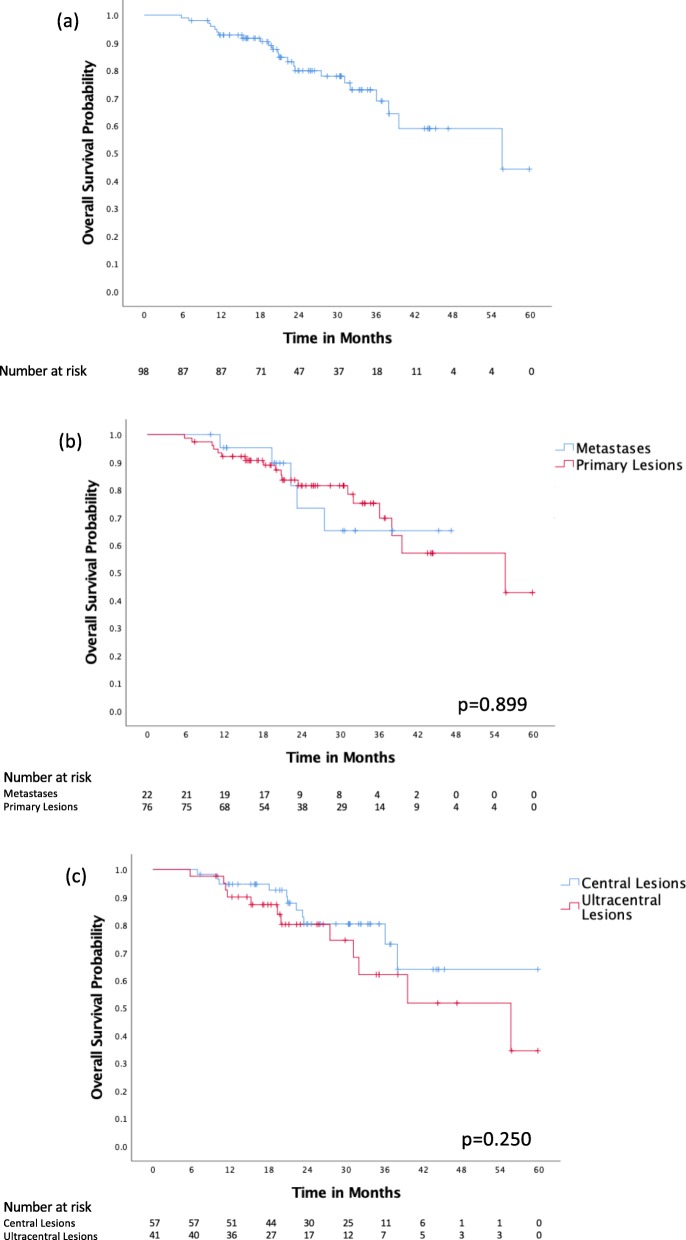
Overall survival for (**a**) all patients, (**b**) primary lesions vs metastases, (**c**) central vs ultracentral lesions

The overall survival outcomes are shown in Fig. 4. At the time of analysis, 23 patients passed away, 11 (47.8%) in the central group and 12 (52.2%) in the ultracentral group. Eighteen (78.3%) events occurred in patients treated for a primary lung lesion and 5 (21.7%) in those treated for a metastasis. The 1-, 2- and 3-year rates were 92.7, 79.8 and 72.9%, respectively. The median overall survival was 55.6 months. There were no significant differences in overall survival between primary lesions and metastases (*p* = 0.899), nor between central and ultracentral cases (*p* = 0.250).

Cox regression analysis was performed to identify factors predictive of local disease control (Table 2). Patient, disease and dosimetric parameters for ITV as well as PTV were included in the univariable analysis. ITV volume (cc) (*p* < 0.001), PTV volume (cc) (*p* < 0.001) and PTV volume receiving < 60 Gy (cc) (*p* < 0.001) were demonstrated to be significant predictors. Factors with a *p*-value < 0.1 on univariable analysis were included in the multivariable analysis. PTV volume (cc) was not included due to collinearity with ITV volume (cc). ITV volume (cc) was the only predictor for local control retaining significance on multivariable analysis (*p* = 0.001).

**Table 2:** Univariable and Multivariable Cox Regression Analysis of Predictors for Local Failure

Variables	Univariable Analysis		Multivariable Analysis	
	HR (95.0% CI)	*p*-value	HR (95.0% CI)	*p*-value
Age at SBRT completion^d^	0.996 (0.938–1.057)	0.889		
Gender				
Male	Reference			
Female	1.483 (0.389–5.662)	0.564		
ECOG performance status				
0–1	Reference			
2–3	1.157 (0.329–4.069)	0.820		
Age-adjusted Charlson comorbidity index ^d^	1.003 (0.726–1.387)	0.983		
COPD				
No COPD	Reference			
GOLD stage 1–2	1.198 (0.318–4.510)	0.789		
GOLD stage 3–4	0.785 (0.140–4.409)	0.783		
Smoking status				
Active smoker	Reference			
Past smoker	0.688 (0.184–2.575)	0.579		
Never smoker	0.598 (0.109–3.292)	0.555		
Tumor location				
Central	Reference			
Ultracentral	0.757 (0.217–2.642)	0.663		
Tumor type				
Primary lung tumor	Reference			
Metastasis	0.759 (0.163–3.536)	0.726		
ITV volume (cc) ^d^	1.049 (1.025–1.074)	< 0.001	1.047 (1.020–1.076)	0.001
ITV Dmin ^a, d^	1.009 (0.974–1.046)	0.607		
ITV Dmax ^a, d^	1.059 (0.969–1.158)	0.205		
ITV Dmean ^a d^	1.025 (0.950–1.106)	0.527		
ITV V 60 Gy ^b, d^	1.037 (0.953–1.128)	0.396		
ITV D 99% ^a, d^	1.003 (0.965–1.043)	0.876		
ITV D 1% ^a, d^	1.044 (0.956–1.141)	0.333		
ITV volume receiving < 60 Gy (cc) ^d^	1.063 (0.894–1.264)	0.488		
PTV volume (cc) ^d, e^	1.029 (1.014–1.044)	< 0.001		
PTV Dmin ^a, d^	1.003 (0.968–1.040)	0.863		
PTV Dmax ^a, d^	1.053 (0.961–1.154)	0.266		
PTV Dmean ^a, d^	1.037 (0.934–1.151)	0.492		
PTV V 60 Gy ^c, d^	1.010 (0.968–1.055)	0.633		
PTV D 99% ^a, d^	1.006 (0.960–1.054)	0.795		
PTV D 1% ^a, d^	1.042 (0.950–1.143)	0.378		
PTV volume receiving < 60 Gy (cc) ^d^	1.044 (1.008–1.081)	0.017	1.006 (0.956–1.058)	0.819

*Abbreviations*: *HR* hazard ratio, *CI* confidence interval, *SBRT* stereotactic body radiotherapy, *COPD* chronic obstructive pulmonary disease, *GOLD* global initiative for chronic obstructive lung disease, *ITV* internal target volume, *Dmin* minimum point dose, *Dmax* maximum point dose, *Dmean* mean dose, *V 60 Gy* volume receiving at least 60 Gy, *D 99%* maximum dose covering 99% of the volume, *D 1%* maximum dose covering 1% of the volume, *PTV* planning target volume

^a^Percentage (%) of the prescription dose of 60 Gy

^b^Percentage (%) of the total ITV volume

^c^Percentage (%) of the total PTV volume

^d^Analysis done using continuous variables

^e^PTV volume (cc) was not included in the multivariable analysis due to collinearity with ITV volume (cc)

### Toxicity

High-grade treatment-related toxicities are outlined in Table 3. Grade 3 events occurred in a total of 5 (5.1%) patients; 3 (5.3%) in the central group and 2 (4.9%) in the ultracentral group. In the central group, 2 (3.5%) patients developed chronic dyspnea requiring home oxygen, occurring 117 and 790 days after completion of SBRT. The latter patient had known underlying idiopathic pulmonary fibrosis and had significant progression of the interstitial lung disease after treatment. One (1.8%) patient with central tumor developed radiation pneumonitis 45 days after treatment and required hospitalization, steroids, and supplemental oxygen. In the ultracentral group, 1 (2.4%) patient developed chronic dyspnea requiring home oxygen 182 days after treatment. One (2.4%) patient developed recurrent episodes of significant hemoptysis, starting 346 days after treatment, and required repeated hospitalizations. No case of grade 4 or grade 5 toxicity was identified in this cohort. There was no high grade esophageal or cardiac toxicity.

**Table 3:** Stereotactic Body Radiotherapy Treatment-Related Toxicities

CTCAE V5 grade 3 toxicity	All patients (*n* = 98)	Central lesions (*n* = 57)	Ultracentral lesions (*n* = 41)
Dyspnea	3 (3.1%)	2 (3.5%)	1 (2.4%)
Pneumonitis	1 (1.0%)	1 (1.8%)	0
Hemoptysis	1 (1.0%)	0	1 (2.4%)
Cough	0	0	0
Esophagitis	0	0	0
Cardiac	0	0	0
Total	5 (5.1%)	3 (5.3%)	2 (4.9%)

*Abbreviation*: *CTCAE V5* common terminology criteria for adverse events version 5.0

## Discussion

We report our institutional outcomes for the SBRT treatment of central and ultracentral lung tumors at a dose of 60 Gy in 8 fractions. As shown in Table 1, the 2 groups are similarly matched, with no significant difference in baseline patient and tumor factors. Our results showed that SBRT treatment of central and ultracentral lung tumors at a dose of 60 Gy in 8 fractions achieves high rates of local control and is associated with excellent survival outcomes. We found low incidences of severe treatment-related toxicities, with no significant difference between central and ultracentral lung tumors.

Although there is no recognized standard dose schedule for the SBRT treatment of central and ultracentral lung tumor, 60 Gy in 8 fractions is frequently used and has been reported in previous series [11, 15, 16]. A retrospective study by Onishi et al. revealed that lower rates of local recurrence (8.1% vs 26.4%, *p* < 0.05) and improved 3-year overall survival (88.4% vs 69.4%, *p* < 0.05) are seen for stage I NSCLC when treated with a biologically effective dose, with an alpha/beta ratio (α/β) of 10 (BED_10_), greater or equal to 100 Gy [17]. Furthermore, a systematic review of central lung tumors by Senthi et al. showed that using fractionation schedules with BED_10_ of 100 Gy or higher and a biologically effective dose with an α/β of 3 (BED_3_) of 210 Gy or less can achieve local control rates greater than 85%, with treatment-related mortality rates of less than 1% [18]. 60 Gy in 8 fractions corresponds to a BED_10_ of 105 Gy and a BED_3_ of 210 Gy, thereby fitting the above criteria.

We used the commonly accepted criteria of the RTOG 0813 study to define central lung tumors [13]. However, ultracentral lung tumor is a newer concept and there is currently a lack of consensus regarding its exact definition. Some of the few series published to date referred to it as a lesion with either gross disease [9, 10, 19] or PTV [20] encompassing the central airways. For our study, we opted for the broader definition used in the ongoing multicentre phase 1 dose escalation SUNSET trial, which also includes cases of PTV overlapping with the esophagus, pulmonary vein or pulmonary artery, as those situations may also significantly increase treatment-related risks [14]. Although, 35 / 41 (85.3%) of the ultracentral cases in our study had PTV encompassing the proximal bronchial tree, with only 6 /41 (14.6%) qualifying solely based on PTV overlapping with the esophagus, pulmonary vein or pulmonary artery.

Our 1-, 2- and 3-year local control rates of respectively 97.8, 93.7 and 84.5% are favorable and on the higher end of the results published to date for the SBRT treatment of central and ultracentral lung lesions [11, 13, 18]. One can also view our results as relatively comparable to the outcomes achieved for the treatment of peripherally situated lesions [2, 21]. Our institution’s earlier outcomes for SBRT treatment of peripheral lesions were reported, with a local control rate of 92% at 2 years [22]. Our results beyond 3 years would be less representative, taking into account the median follow-up time of 22.9 months and the limited number of cases remaining past that time point. Similar to reports by Raman et al. [16] and Chang et al. [23], we did not detect a significant difference in local recurrence rate between the central and ultracentral groups (*p* = 0.662). Patients with ultracentral lesions did not have inferior local control outcomes despite having poorer ITV and PTV dose coverage. (Fig. 1).

As depicted in Fig. 1 and Table 2, we performed a comprehensive analysis of the SBRT treatments’ dosimetric parameters, with the aim of identifying predictors for local control outcomes. Only ITV volume remained a significant factor on multivariable analysis (*p* = 0.001). A study by Modh et al. echoes our results, having identified the GTV size as the sole significant predictor of local control outcomes for central lung tumors (*p* = 0.03) [24]. PTV volume was also identified as a significant predictor on univariable analysis but was not included in the multivariable analysis, considering it is collinear and proportional to ITV volume. However, the PTV volume may potentially have a similar predictive impact on local disease control outcomes.

For the treatment of central and ultracentral lung lesions, it is controversial whether one should prioritize the OARs dose constraints over the target volumes coverage, or vice-versa. In our institution, minimizing excessive dose to critical OARs takes precedence. This is reflected by the fact that 45 (45.9%) of our cases had compromised target volumes coverage. We did not identify a statistically significant difference in local control for patients who had both compromised ITV and PTV coverage (*n* = 16, 16.3%), compromised PTV coverage only (*n* = 29, 29.6%) or optimal target volume coverage (*n* = 53, 54.1%). Although, we do observe a possible trend to worse local control outcomes for patients with both compromised ITV and PTV dosimetric coverage, as they had poorer local control rates at the 24 months mark. (Fig. 3) We also analyzed dosimetric parameters that can be reflective of potential compromised target volumes coverage. Notably, the ITV and PTV Dmin, ITV and PTV D 99% and ITV volume receiving less than 60 Gy were not found to be significant on univariable analysis. While the PTV volume receiving less than 60 Gy was significant on univariable analysis, it did not retain its significance on multivariable analysis. Coupled with our favorable local control outcomes, these results potentially support our practice of prioritizing OARs constraints.

Our overall survival rates of 92.7, 79.8 and 72.9% at respectively 1, 2 and 3 years, with a median of 55.6 months, are also excellent in comparison to those in other published central and ultracentral lung SBRT studies [13, 16, 23]. We found comparable overall survival outcomes between the groups of central and ultracentral lung tumors (*p* = 0.250), similar to findings reported in other series [16, 23].

We detected low rates of severe treatment-related toxicities, with a total of 5 (5.1%) patients developing grade 3 events, comparable between the central (3 / 57, 5.3%) and ultracentral (2 / 41, 4.9%) groups. There were no grade 4 or 5 events. A few other studies available in the literature also demonstrated low risks for severe toxicities. A series by Haasbeek et al. reported the outcomes of 63 patients with central lung tumors treated with 60 Gy in 8 fractions. Their aim was to have 99% of the PTV covered by the prescription dose, without any deliberate underdosing of the target volumes [11]. There were 6 (9.5%) cases of grade 3 toxicities, and no grade 4 or 5 events. The authors however did not specify whether any of the treated lesions could have met the criteria for ultracentral. A study by Raman et al. reported the outcomes of patients with central and ultracentral lesions treated with various dose schedules [16]. Among them, 91 patients received the 60 Gy in 8 fractions regimen, with only 1 (1.1%) case of grade 3 pneumonitis reported in the central group. There were also no grade 4 or 5 events. Their institutional practice was to compromise target volume coverage to meet OARs constraints. In contrast, several other studies reported higher incidences and more severe treatment-related toxicities. Notably, the RTOG 0813 phase I/II trial examined at the outcomes of centrally located NSCLC treated in 5 fractions [13]. The PTV coverage was prioritized over the OARs constraints. Four out of 38 (10.5%) patients treated at a dose of 57.5 Gy and 4 / 33 (12.1%) patients treated with 60 Gy developed grade 3 or higher toxicities during the first year following SBRT completion. Beyond the first year, treatment-related death occurred in 3 (7.9%) patients in the 57.5 Gy arm and 1 (3.0%) in the 60 Gy arm. Tekatli et al. studied the outcomes of 47 patients with ultracentral lung tumors treated with 60 Gy in 12 fractions and reported a 38% rate of grade 3–5 toxicities, with 10 (21%) of patients having treatment-related death [20]. In this study, a maximum dose up to 140% of the prescription was allowed. Twenty-five (53%) patients had endobronchial tumor, which is a risk factor for pulmonary hemorrhage in the context of radiotherapy treatment [25]. This was identified as the cause of death for 7 (15%) of the patients.

Moreover, a study by Stam et al. demonstrated that patients who underwent SBRT to central lung tumors located within the first centimeter surrounding the proximal bronchial tree were more likely to die from causes other than cancer compared to other patients [26]. The authors detected that high dose to the proximal bronchial tree (D 1%) was significantly associated with noncancer death (*p* = 0.003). Our study’s favorable toxicity profile may be attributed to the protracted fractionation schedule of 60 Gy in 8 fractions and the practice of minimizing excessive overdosage of the critical OARs by sacrificing target volume coverage when a compromise is required. However, considering our median follow-up time of 22.9 months, there may still be potential risks for undetected later toxicities. Of note, 1 (1.0%) case of late grade 3 dyspnea occurred in a patient with underlying interstitial lung disease, which is a well-established predictor for worse SBRT-related toxicity [27, 28]. Considering the small number of high-grade events, an analysis for the predictors of toxicity could not be meaningfully performed.

To our knowledge, this is the largest study to date reporting the outcomes of SBRT at a dose of 60 Gy in 8 fractions for the treatment of central and ultracentral lung tumors. Our results support the use of this dose schedule while prioritizing OARs constraints, as the practice is associated with high rates of local control and excellent overall survival, as well as low rates of severe toxicities. A dosimetric ultracentral lung SBRT study by Murrel et al. further supports our findings [29]. The authors analyzed regimens of 50 Gy in 5 fractions, 60 Gy in 8 fractions and 60 Gy in 15 fractions, either prioritizing OARs constraints or PTV coverage. They concluded that the treatment at 60 Gy in 8 fractions prioritizing OARs would be advisable, as it yielded the most acceptable balance between local control and toxicity, with a calculated tumor control probability of 65.7% and a risk of major pulmonary complication of < 1%.

In terms of potential limitations, this study carries the inherent biases associated to its retrospective nature. As the patients who underwent treatment were non-randomly selected, there may be a potential selection bias. The patients with central and ultracentral lung tumors to whom were offered SBRT treatment may inherently have superior outcomes. There may also be a bias attributed to non-random loss to follow-up, as patients experiencing adverse events or recurrent disease may be more at risk to be lost. Also, the median follow-up time is 22.9 months which can be viewed as relatively short. However, this duration is likely sufficient for the analysis of treatment effectiveness, as a large retrospective study looking at patterns of disease recurrence after lung SBRT reported median times to local, regional and distant recurrences of respectively 14.9, 13.1 and 9.6 months [21]. Nonetheless, although most cases of late toxicities would occur within 18 months of treatment completion, toxicities developing after 2 years have previously been reported [20]. Moreover, the relatively small number of detected local failure events, 11 in the entire cohort, may have limited our power to detected statistically significant differences and predictors of outcomes. Furthermore, we did not detect any difference in local control rates and overall survival outcomes between primary lung lesions and metastases. However, only 22 (22.4%) patients in this study were treated for metastatic disease; therefore, this may have limited our ability to detect potential subtle differences in treatment outcomes among diverse patient subsets. Finally, all patients in our cohort underwent SBRT with motion encompassing method, with ITV including tumor movement on all phases of the 4DCT scan. Active motion management techniques (abdominal compression, respiratory gating, breath hold and dynamic tumor tracking) were systematically not used. Whether these techniques may lead to clinically impactful improvement in target volumes and OARs dosimetry as well as measurable reduction in toxicity for the treatment of central and ultracentral lung tumors would be an important topic for further research.

## Conclusions

This study demonstrates the efficacy and safety of SBRT at a dose of 60 Gy in 8 fractions for the treatment of central and ultracentral lung tumors, with an approach of compromising target volumes coverage to minimize excessive dose to critical OARs. There was no significant difference in local control, overall survival and high-grade toxicity outcomes between the central and ultracentral lung tumors groups.

## Supplementary information

**Additional file 1.** Appendix A: Lung SBRT 60 Gy in 8 fractions planning dose constraints [30].

## Data Availability

The data that support the findings of this study are available from the corresponding author, but restrictions apply to the availability of these data, which were used under license for the current study, and so are not publicly available. Data are however available from the authors upon reasonable request and with permission of University of British Columbia - British Columbia Cancer Agency Research Ethics Board.

## Abbreviations

3DCRT: 3-dimensional conformal radiotherapy
4DCT: 4-dimensional computed tomography
AAA: Analytical Anisotropic Algorithm
C: Central
CBCT: Cone beam computed tomography
CI: Confidence interval
COPD: Chronic obstructive pulmonary disease
CT: Computed tomography
CTCAE: Common terminology criteria for adverse events
D 1%: Maximum dose covering 1% of the volume
D 99%: Maximum dose covering 99% of the volume
Dmax: Maximum point dose
Dmean: Mean dose
Dmin: Minimum point dose
ECOG: Eastern cooperative oncology group
GOLD: Global initiative for chronic obstructive lung disease
GTV: Gross tumor volume
HR: Hazard ratio
IMART: Intensity modulated radiotherapy
ITV: Internal target volume
KV: Kilovoltage
LC: Local control
NSCLC: Non-small cell lung cancers
OARs: Organs at risk
PET: Positron emission tomography
PTV: Planning target volume
RECIST: Response evaluation criteria in solid tumors
RTOG: Radiation Therapy Oncology Group
SBRT: Stereotactic body radiotherapy
UC: Ultracentral
V 100%: Volume receiving at least 100% of the prescription dose
V 60 Gy: Volume receiving at least 60 Gy
V 90%: Volume receiving at least 90% of the prescription dose
VMAT: Volumetric modulated arc therapy
α/β: Alpha/beta ratio
