# Supplementary material for: Outcomes of stereotactic body radiotherapy 60 Gy in 8 fractions when prioritizing organs at risk for central and ultracentral lung tumors
Source: Radiat Oncol. 2020 Feb 27;15:50. doi: 10.1186/s13014-020-01491-w (PMC7047404; doi:10.1186/s13014-020-01491-w)
Supplement: Supplementary file 1 — Additional file 1. Appendix A: Lung SBRT 60 Gy in 8 fractions planning dose constraints [30]. [file 13014_2020_1491_MOESM1_ESM.docx]

**Appendix A**

| Lung SBRT 60 Gy in 8 fractions Planning Dose Constraints | |
| --- | --- |
| Spinal cord  Dmax | < 30.6 Gy |
| Proximal bronchial tree  D0.035 cc^1^ | < 46.3 Gy |
| Proximal trachea  D0.035 cc^1^ | < 46.3 Gy |
| Esophagus  D0.035 cc^1^ | < 40 Gy |
| Great vessels  D0.035 cc^1^ | < 65 Gy |
| Brachial plexus  D0.035 cc^1^ | < 35 Gy |
| Heart  D0.035 cc^1^  V 39 Gy | < 46 Gy  < 15 cc |
| Both lungs  Dmean  V 26 Gy  > 1500 cc normal lung | < 7 Gy  < 10%  < 14 Gy |
| Chest wall^2^  D0.035 cc^1^  V 45 Gy | < 68 Gy  < 30 cc |
| ^1^ Use of D0.035 cc consistent to the recommendations from American Association of Physicists in Medicine (AAPM) Task Group 101 report [30]  ^2^ Chest wall constraint prioritized below PTV coverage  Abbreviations: Dmax = absolute maximum point dose, D0.035 cc = maximum dose to 0.035 cc, V 39 Gy = volume receiving at least 39 Gy, Dmean = mean dose, V 26 Gy = volume receiving at least 26 Gy, V 45 Gy = volume receiving at least 45 Gy. | |
